# Concluding remarks for *Faraday Discussion* on Water at Interfaces

**DOI:** 10.1039/d3fd00153a

**Published:** 2023-12-15

**Authors:** Mischa Bonn

**Affiliations:** a Max Planck Institute for Polymer Research Ackermannweg 10 55128 Mainz Germany bonn@mpip-mainz.mpg.de

## Abstract

Water at interfaces is a fascinating and multifaceted topic that has garnered significant attention in various scientific fields due to its relevance and implications. This *Faraday Discussion* explored the complexity of water at different interfaces. Many of the reports highlight the need for a molecular-level understanding. The *Discussion* was lively and constructive. In these summarizing remarks, I do not aim to be complete, but will rather try to sketch the status of the field, highlight the progress that we as a community have made, and present eclectic examples of where more work needs to be done.

## Abstract 2

This *Faraday Discussion* explored the complexity of water at different interfaces. These summarizing remarks sketch the status of the field, highlight the progress that the community has made, and present eclectic examples of where more work needs to be done.

## Graphical abstract

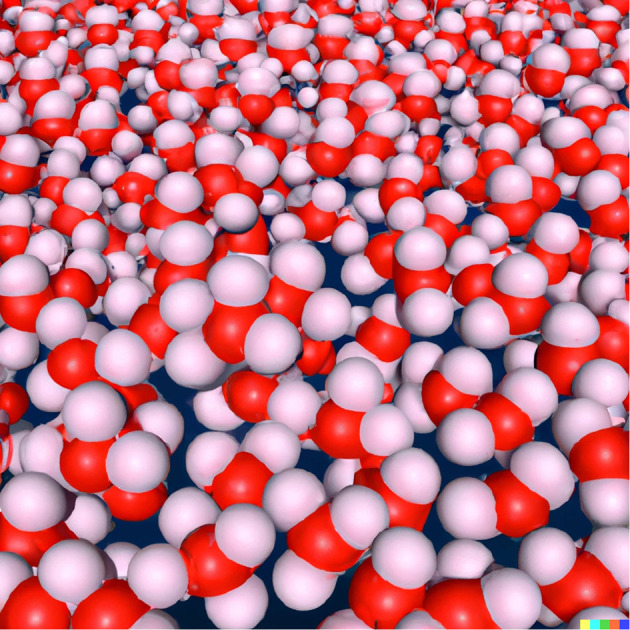

The “Water at Interfaces” *Faraday Discussion* brought some of the leading experts in the field together, both virtually and in-person in London. Interestingly, there was a previous *Faraday Discussion* (No. 141) in 2008, entitled: “Water – From Interfaces to the Bulk”, giving us a unique opportunity to explore the progress made in the past 15 years. This comparison, I submit, shows the amazing progress in some areas and the sobering realization that crucial open questions remain in some others.

For the good news:

First of all, we are no longer arguing about the quality of different water models (we were in 2008): theory has come so far! To highlight some achievements in recent years: firstly, the TIP4P/2005 and TIP4P/ice models seem to provide excellent descriptions of water and ice consistently. The limitation of these models is that the water model is rigid and not polarizable. Successful models that include polarization effects are poli2vs,^1^ specifically aimed at reproducing the vibrational spectroscopy of water, and MB-pol,^2^ which provides good agreement with thermodynamic observables across a wide temperature range. An orthogonal approach, namely to simplify, rather than expand, the water model, was taken by Molinero and Moore.^3^ In their mW model, water is portrayed as an atom with tetrahedrality that is intermediate between carbon and silicon. Despite relying solely on short-range interactions, mW accurately replicates the energetics, density, and structure of liquid water. The accelerated computational efficiency offered by mW allows for investigating slow processes in, for instance, deeply supercooled water, including in ice nucleation. Finally, density functional theory molecular dynamics (DFT-MD), with the support of machine learning, is the most recent advance,^4^ and has enabled, for instance, the predicting of the phase diagram of monolayer nanoconfined water.^5^

These advances in modeling, along with those in experiments, now allow for a direct, quantitative comparison of complex aqueous systems (*e.g.*, density functional theory and atomic force microscopy on well-defined surfaces in ultrahigh vacuum): theory and experiment have really come together, as evident from several papers in this present issue of *Faraday Discussions*††https://doi.org/10.1039/D3FD00097D, https://doi.org/10.1039/D3FD00095H, https://doi.org/10.1039/D3FD00093A, https://doi.org/10.1039/D3FD00140G, https://doi.org/10.1039/D3FD00099K, https://doi.org/10.1039/D3FD00111C, https://doi.org/10.1039/D3FD00113J, https://doi.org/10.1039/D3FD00100H, https://doi.org/10.1039/D3FD00110E and https://doi.org/10.1039/D3FD00102D. that have theory directly connecting to experiments, or *vice versa*.

A second – heated – debate that has been ongoing revolves around the distribution of ions near or at interfaces in ionic solutions, which is crucial, especially in the context of ocean acidification. The dissolution of excess carbon dioxide from the atmosphere into the oceans can alter the pH and affect the stability of biominerals. The distribution of ions near the interface plays a significant role in these processes and can impact marine life, including organisms that rely on calcium carbonate to build their shells and skeletons. Likewise, the ion distribution at the water surface is important for (photo-)chemistry occurring on the surface in the atmosphere.

In the 2008 *Faraday Discussion*, a major discussion emerged about the surface activity of hydrated protons or hydroxyl ions. From the 2008 Spiers Memorial Lecture: ‘Therefore, in the topmost layer of water there shall be an excess of hydronium over hydroxide. We denote this situation as an “acidic” surface of water’.^6^ Yet, the title of the subsequent paper from the same *Faraday Discussion* reads: “The surface of neat water is basic”.^7^ Meanwhile, this controversy has been resolved by quantitative measurements of proton and hydroxyl propensity at the water–air interface. There is indeed an excess of hydronium at the surface, while hydroxide ions are depleted from the surface. There is an adsorption well for hydronium at the water–air interface, which has been quantified independently by two groups^8,9^ as being characterized by an adsorption free energy, Δ*G* ≈ −4.5 kJ mol^−1^, corresponding to a partitioning coefficient of the surface with respect to bulk water of a factor of ∼5. Such a surface propensity is absent for hydroxyl ions.^10^ As highlighted in this year’s Spiers Memorial Lecture, we now also have a much better understanding of how other ions behave at the water–air interface,^11^ and how we can use ion exchange to enable micro swimmers in water (https://doi.org/10.1039/D3FD00098B), for instance.

Nanoconfined water was already a hot topic (featuring in 5 out of ∼20 papers) in 2008.^12–16^ But in 2023, we have much better control over confinement^17^ using advanced nanofabrication techniques and much more advanced measurement methods^18^ for understanding nanoconfined water, and we can even study non-equilibrium effects in nanoconfined water (*e.g.*, ‘quantum friction’) (https://doi.org/10.1039/D3FD00115F, ).^19^

We know much more about (the subtleties of) biomolecular hydration. Hydration of biomolecules appears to be at least as complex as water itself.^20^ Subtle and individual effects occur in the hydration of generally non-polar solutes in water (https://doi.org/10.1039/D3FD00104K and https://doi.org/10.1039/D3FD00109A),^21^ and specifically hydrated DNA (https://doi.org/10.1039/D3FD00109A),^21^ proteins,^22^ and phospholipids (https://doi.org/10.1039/D3FD00117B, https://doi.org/10.1039/D3FD00094J and https://doi.org/10.1039/D3FD00118K). While not everybody will agree with this statement, it seems increasingly apparent that biomolecular hydration is a highly local effect, and water structuring is limited to the first 1–2 layers of water molecules around the biomolecules. Yet the precise nature of this structuring depends critically on the biomolecule’s characteristics.

And now, the sobering awareness that essential unanswered questions persist:

Despite the enormous progress that the community has clearly made, one can also identify remaining open questions in areas where more research is needed. Somewhat ironically, an important interface that still raises controversial and open questions is the water–air interface. Despite arguably being the simplest aqueous surface conceivable, there is considerable debate about whether this nominally neutral interface is, in fact, neutral. One intriguing phenomenon is the enhanced chemistry occurring on the water surface, compared to reaction rates in the bulk. Somewhat controversial^23^ in this context are the claims of spontaneous dissociation of water molecules at the surface of pure water microdroplets.^24^ This dissociation is thought to be induced by a large interfacial electric field leading to the formation of OH radicals. These radicals can subsequently combine to form hydrogen peroxide (H_2_O_2_) through an associative reaction. Strong electric fields have been reported by indirect measurements,^25^ and one of the challenges is clearly to measure these field strengths more directly, for instance, using nonlinear optical approaches.^26^

In various environmental, biological, and technological contexts, water interfaces with charged surfaces. Fundamental questions remain about water and aqueous electrolyte solutions at charged interfaces. These questions pertain to concepts like the Debye length, the magnitude of the interfacial electric field and associated surface potentials, and related to this, fundamental questions about ion–water, ion–ion, and ion–surface interactions. Indeed, much like the situation in 2008,^27^ several papers in the 2023 edition of this *Faraday Discussion* address the behavior of water at charged interfaces.‡‡https://doi.org/10.1039/D3FD00114H, https://doi.org/10.1039/D3FD00103B, https://doi.org/10.1039/D3FD00107E, https://doi.org/10.1039/D3FD00133D and https://doi.org/10.1039/D3FD00124E.

## Conclusions

Finally, there was a recurrent discussion during the meeting about the definition of *the* water interface. That debate reminded me of a quote from Robert M. Pirsig^28^ about quality: “Quality. You know what it is, yet you don’t know what it is. … But for all practical purposes it really does exist.”. Along the same lines, I would argue that the interface most certainly exists, even if we cannot define it unequivocally. What is more, it has wonderful people of outstanding quality studying it.

## Author contributions

M. B. wrote and modified the manuscript.

## Conflicts of interest

There are no conflicts to declare.

## Supplementary Material
